# Rolling Contact Fatigue Behavior of Pitch Bearing Raceway in Offshore Wind Turbines

**DOI:** 10.3390/ma18081816

**Published:** 2025-04-15

**Authors:** Haifeng He, Yiming Chen, Yang Liu, YongChao Zhu, Xin Jin

**Affiliations:** 1 School of Mechanical and Electrical Engineering, Guizhou Normal University, Guiyang 550025, China; 2 Taihang Laboratory, Chengdu 610000, China; 3 Department of Mechanics, Tsinghua University, Beijing 100084, China; 4 China Gas Turbine Establishment, Chengdu 610500, China; 5 State Key Laboratory of Mechanical Transmission for Advanced Equipment, Chongqing University, Chongqing 400044, China

**Keywords:** offshore wind turbine, pitch bearing, rolling contact fatigue (RCF), continuum damage, life prediction

## Abstract

As critical components in offshore wind turbine (OWT) systems, pitch bearings require exceptional fatigue resistance to ensure the extended operational lifespan and structural reliability demanded by marine environments. Failure of these bearings due to rolling contact fatigue (RCF) can severely affect the economic efficiency of offshore wind turbines and potentially lead to safety accidents involving both humans and machinery. A simulation model for pitch bearings used in a 3 MW OWT is established to study the RCF behavior under operational conditions based on continuum damage mechanics. Both the elastic and plastic damage are considered in the damage process through a Python script. A user subroutine UMAT is programmed to depict the gradual deterioration of mechanical properties. The results indicate that the fatigue damage of the raceway exhibits significant nonlinear characteristics, with elastic damage playing a predominant role in determining its fatigue life under operational conditions.

## 1. Introduction

Offshore wind power has seen tremendous development due to high wind speed, high number of power generation utilization hours, and the fact that it does not occupy land. As the designed generation power and lifespan of offshore wind turbines (OWTs) continue to increase, the service performance requirements for one of their core components, the pitch bearings, have become more stringent. The main failure moddes of pitch bearings include lubrication failure [1], improper assembly [2], plastic deformation [3], and rolling contact fatigue (RCF). Among these, RCF failure occurs frequently in OWT pitch bearings in practical engineering applications. The RCF failure of bearings is influenced by several factors, such as loading conditions [4], frictional states [5], water contaminations [6], and materials [7,8]. In recent years, various scholars have tried to investigate the bearing RCF from different perspectives.

In the experimental studies, the white etching area and dark etching area, as the typical forms of RCF, were deeply investigated by several researchers [9,10,11]. The three-ball-rod RCF tests were conducted by Guo et al. [12], and they found that the dark etching area was located in the region in which the shear stress was maximum. Chen et al. [13] simulated the formation of the white etching area during the RCF process based on a thermodynamically consistent model. Su et al. [14] assumed that the white etching area is induced by the coupled effect of shear plastic deformation and RCF. Morsdorf et al. [15] analyzed the evolution of the white etching area for wind turbine bearings in the RCF process. In addition, the effects of treatment on the RCF of bearings were investigated. Zhu et al. [16] experimentally studied the RCF behavior of bearings under the surface ultrasonic rolling process (SURP). It was found that the SURP benefits the RCF performance through enhancing surface compressive residual stress and reducing the roughness. Cao et al. [17] experimentally studied the effect of vacuum carburization surface treatment on the RCF life of 100Cr6 bearing steel. The fatigue lifespan grew from 10 million cycles to 100 million cycles, a 10-fold growth, after the enhancement. Cai et al. [18] experimentally found that direct cryogenic treatment after quenching for M50 bearing steel benefits the fatigue performance. Yang et al. [19] studied the influence of rare earth on the RCF behavior of M50 bearing steel through ball-on-rod experiments. The L10 RCF life increased by 96.2% after the addition of rare earth. Liang et al. [20] found that the hydrogen in the mechanical components had a negative effect on RCF life through the ball-on-rod fatigue test. Qu et al. [21] experimentally proved that the diffraction peak width is an indicator for the bearing RCF state through the X-ray diffraction method. Based on the triple-disc contact fatigue test, Kunzelmann et al. [22] investigated the crack growth path and rate for an AISI 52100 bearing steel roller. Subsequently, the experimental data were applied to establish a crack propagation model for RCF.

The experimental tests are time consuming and labor intensive, which, to some extent, limits in-depth research on bearing RCF behavior. With the development of computer science and technology, fatigue simulation has provided an effective way for bearing RCF analysis. Shao et al. [23] assumed that the internal defects determined the overall performance of high-strength steel and proposed an RCF initiation model to study the impact of internal defects on the fatigue process for bearing steel based on the shakedown theory. Wang et al. [24] proposed a coupled mechanical-diffusion peridynamic (PD) fatigue model to analyze the formation of white etching cracks for bearing steel in the RCF process. The effects of internal defects and contact states on the white etching cracks were studied. Ravi et al. [25] simulated RCF crack initiation around the area of non-metallic inclusions for bearing steel. In the prediction on bearing RCF lifespan, Foko et al. [26] predicted the RCF life of the type NU208 roller bearings for different surface roughness states through the Fatemi–Socie criterion. Yu et al. [27] proposed a modified Smith–Watson–Topper model considering the mean stress correction to predict the fatigue life of rolling bearings. Menck [28] calculated the RCF life of rolling bearings under stochastic operating conditions through the finite segment method. Vijay and Sadeghi [29] proposed a crystal plasticity and cohesive element model to predict the crack initiation and crack propagation life for rolling bearings. Zhao et al. [30] proposed a life prediction model to investigate the effects of gradient characteristics including the hardness gradient and the gradient structure on the RCF life of bearing steel GCr15 based on the Weibull theory. The predicted results were compared with the existing experimental data. 

According to continuum damage mechanics (CDM) [31], Ma et al. [32] proposed a damage model to predict the RCF life of AISI 52100 bearings used in a high-speed railway. Lorenz et al. [33,34,35] established bearing RCF models to investigate the effects of the spatial hardness distribution and the grain refinement on the bearing RCF behavior. He et al. [36] proposed a damage-coupled model to investigate the influence of ultimate loading conditions on the RCF life for a pitch bearing raceway used in OWTs. Cu and Su [37] proposed an RCF model to predict the early fatigue failure of bearings considering the surface integrity based on the CDM method. The fatigue lives for different surface roughnesses and hardnesses were also analyzed.

During the pitch bearings’ service process, the mechanical properties of the pitch bearing material gradually deteriorate, and the damage gradually accumulates, ultimately leading to the RCF failure. The previous works have primarily focused on the prediction of the RCF life or the RCF failure modes of pitch bearings. However, the evolutions of the stress response and the material mechanical properties during the service process were hardly reported. And the process of material deterioration is a critical component in shedding light on the RCF failure mechanisms. In order to fill this gap, a damage-coupled simulation model for pitch bearings used in a 3 MW OWT is established to study the RCF behavior under operational conditions based on CDM. The gradual accumulation of damage and deterioration of mechanical properties were revealed in this work.

## 2. Finite Element Model and Damage-Coupled Behavior

### 2.1. Finite Element Model

**Figure 1:**
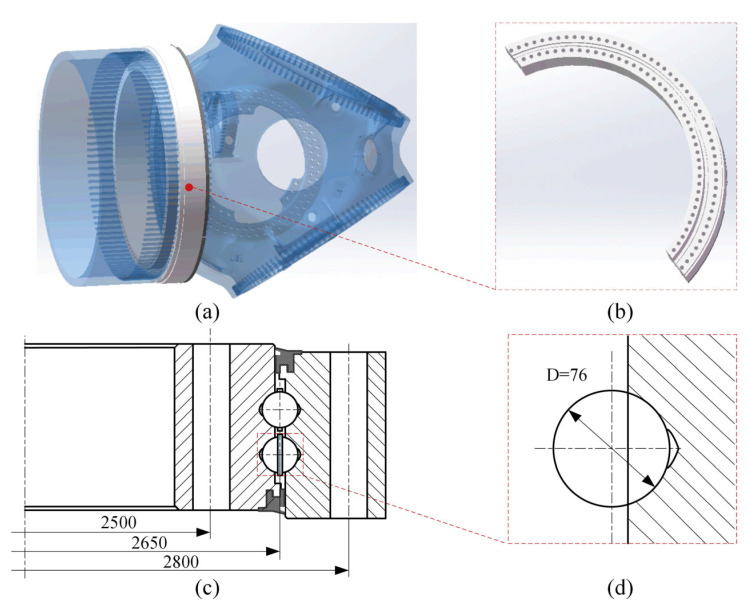
The 3D geometric structure and the 2D model of the pitch bearing; (**a**) the geometric structure of a wind turbine; (**b**) the 3D geometric structure of the pitch bearing; (**c**) the 2D geometric structure of the pitch bearing; (**d**) the contact condition between the roller and the outer ring raceway.

The pitch bearing used originated from a 3 MW offshore wind turbine. Figure 1a shows the geometric structure of this wind turbine, which included three blades and three pitch bearings. Figure 1b and Figure 1c display the 3D geometric structure and the 2D model of the pitch bearing, respectively. The pitch bearing is a double row four-point contact ball bearing. The pitch bearing had an outer ring, an inner ring, and rollers. Each bearing contained 92 rollers. The diameters of the outer ring pitch, the inner ring pitch, and the roller pitch were 2800 mm, 2650 mm, and 2500 mm, as shown in Figure 1c. The diameter of the rollers was 76 mm. Figure 1d shows the contact condition between the roller and the outer ring raceway. During the working process, each roller contacted the outer raceway at two distinct contact points to transfer the applied load.

**Figure 2:**
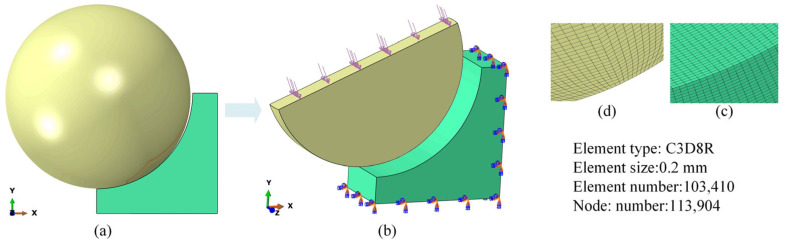
The simulation model for roller–raceway contact; (**a**) the half-contact model between the roller and the bearing raceway; (**b**) the equivalent finite element model; (**c**,**d**) the mesh detail around the contact area.

All the rollers exhibited identical loading histories throughout the service process, resulting in a symmetrical stress distribution pattern. Hence, a simplified equivalent finite element model of the roller-pitch bearing raceway was established to analyze the fatigue behavior of the bearing inner raceway. Figure 2a shows a half-contact model between the roller and the bearing raceway according to symmetry features. Figure 2b displays the equivalent finite element model considering the loading and boundary conditions. Figure 2c,d show the mesh detail around the contact area. The element type for the roller and raceway was chosen as C3D8R to balance precision and computational cost. The minimum mesh size in the contact area was 0.2 mm. The total element and node numbers were 103,410 and 113,904, respectively. A gradually large mesh away from the contact area was applied to reduce the simulation time. The operation load was 159,529 N. Python script v3.9 was used for the finite element simulation including the pre-processor module (Step, Load, and Mesh sections) and post-processor module.

The pitch bearing raceway was produced from 42CrMo4 material. Table 1 lists the chemical composition of the pitch bearing raceway. The residual stress for the pitch bearing was measured as −168 MPa through an X-ray RS diffractometer (Pulstec Industrial Co., Ltd, Tokyo, Japan). And the surface and core hardness were measured as 653 HV and 420 HV using an MHVS-1000A micro-hardness tester (Shanghai Aolong Xingdi Testing Instrument Co., Ltd., Shanghai, China). The case-hardened depth was 4.4 mm. The material of the rollers was GCr15, used in this pitch bearing. The roller was set as pure elastic considering that its mechanical properties were better than that of the raceway material.

### 2.2. Damage-Coupled Model for Pitch Bearing

Damage accumulation will lead to the deterioration of material mechanical properties. The damage-coupled constitutive equation of the pitch bearing is derived as follows:

(1) $$\sigma_{ij}=C_{ijkl}{(1-D)\varepsilon }_{kl}$$

where $\sigma_{ij},$ $C_{ijkl},$ and $\varepsilon_{kl}$ are the stress tensor, the fourth-order elastic tensor, and the strain tensor, respectively. D is the accumulated damage for each material point. The damage ranges from 0 to 1, which represents the material point changes from the intact state to the total failure state.

The damage evolution equation considering both the elastic ($D^{e})$ and plastic ($D^{p})$ parts is given as follows [38,39]:

(2) $$\left(\frac{dD}{dN_{c}}\right)=\left(\frac{dD^{e}}{dN_{c}}\right)+\left(\frac{dD^{p}}{dN_{c}}\right)$$

Both the material hardness and the residual stress have a significant effect on bearing contact fatigue life; hence, the damage rate considering RS and hardness is calculated as follows [40]:

(3) $$\frac{dD^{e}}{dN_{c}}=[\frac{\Delta \tau }{\tau_{R}(1-\frac{\sigma_{r}}{S_{us}})(1-D)}{]}^{m}$$

(4) $$\frac{dD^{p}}{dN_{c}}=[\frac{\sigma_{eq}^{2}}{2ES(1-\frac{\sigma_{r}}{S_{us}}{)}^{2}(1-D{)}^{2}}{]}^{q}\dot{p}$$

where, $\tau_{R}$, m, S, and q are the damage-related parameters. $\sigma_{r}$ and $S_{us}$ are the residual tress and the material ultimate shear strength, respectively. Δτ and $\sigma_{eq}$ are the shear stress range and von Mises stress, respectively. $\tau_{R}$, m, S, and q can be calculated by combining the integration of the damage rate equations and the Basquin law [40]. Table 2 lists the corresponding damage-related parameters of the pitch bearing. The damage-coupled constitutive equations and the damage equations were applied in the finite element model through the user subroutine UMAT in the commercial software ABAQUS v2022. The yield stress $\sigma_{y}$ for the pitch bearing raceway is expressed as follows:

(5) $$\sigma_{y}=2.876HV-90.7$$

**Figure 3:**
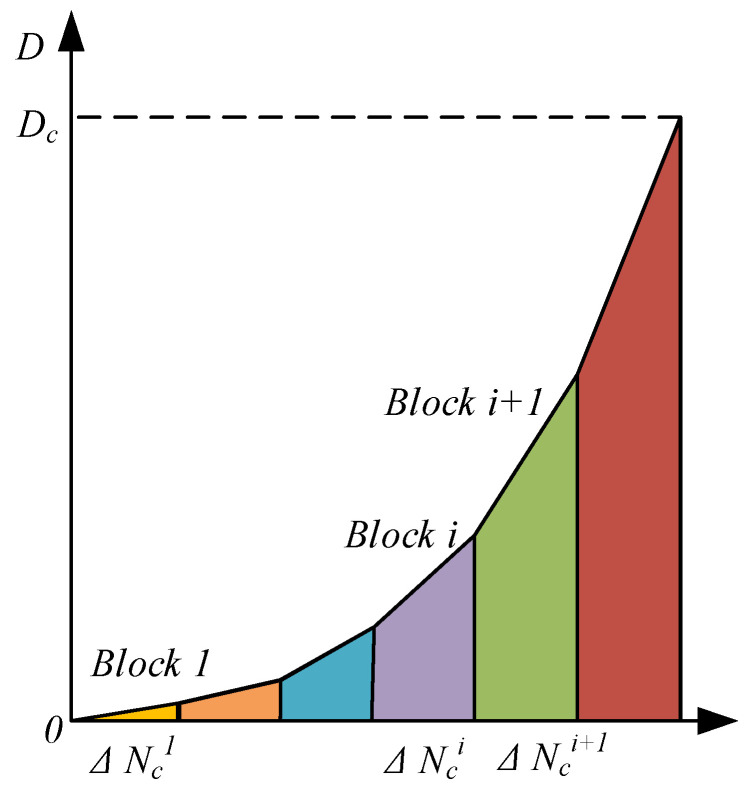
Damage accumulation using the “jump-in-cycles” method.

The designed fatigue life of a pitch bearing is more than a million loading cycles, which is difficult to simulate in ABAQUS v2022. Hence, the “jump-in-cycles” method [41] is used to accelerate the simulation process, as shown in Figure 3. It is assumed that the stress and strain fields remain constant during a $\Delta N_{c}$ loading cycle (which is expressed as a loading block). The damage rate in the constant $\Delta N_{c}$ remains the same accordingly. When damage accumulates to the critical value 1, fatigue failure occurs, and the corresponding loading cycle is the fatigue life.

## 3. Results and Discussion

### 3.1. Stress Response Evolution of Pitch Bearing

**Figure 4:**
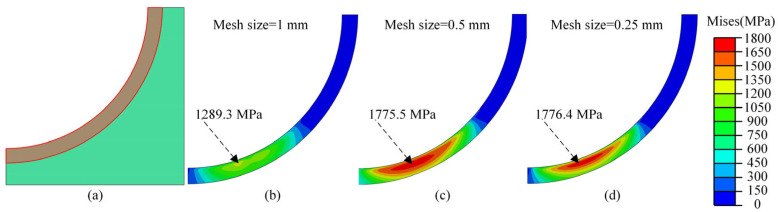
The von Mises stress for different minimum mesh sizes; (**a**) the fine mesh area; (**b**) the von Mises stress for mesh size 1 mm; (**c**) the von Mises stress for mesh size 0.5 mm; (**d**) the von Mises stress for e mesh size 0.25 mm.

Figure 4 shows the von Mises stress contours for various minimum mesh sizes. The results indicate that the stress response escalated as the minimum mesh size diminished, eventually converging to a specific value. Notably, the maximum von Mises stress increased from 1289.3 MPa to 1775.5 MPa and 1776.4 MPa as the minimum mesh size decreased from 1 mm to 0.5 mm and 0.25 mm, respectively. These findings indicate that the stress responses for minimum mesh sizes of 0.25 mm and 0.5 mm exhibit equivalent precision. Consequently, to optimize computational efficiency while maintaining solution accuracy, a minimum mesh size of 0.5 mm was selected in the present study.

**Figure 5:**
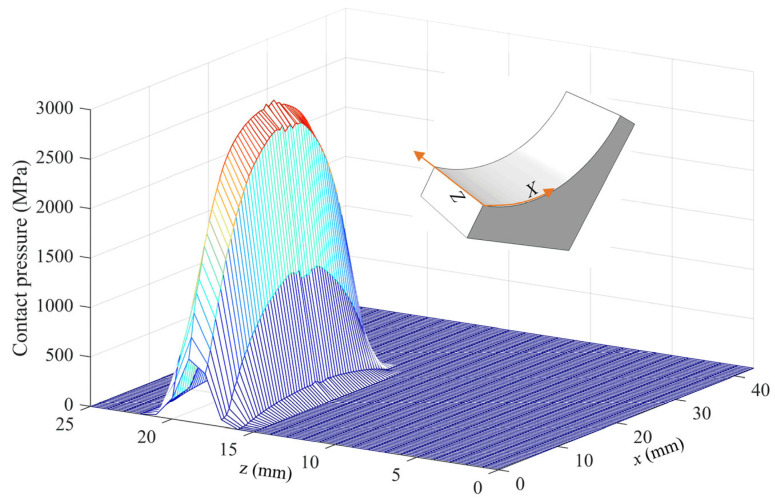
The contact pressure when the raceway is loaded through the roller.

Figure 5 shows the contact pressure when the raceway was loaded through the roller. The contact pressure was highly concentrated in a very small area; the contact widths in the *x* and *z* (the rolling direction) directions were 27 mm and 6 mm, respectively. The contact width in the *z*-direction was significantly smaller than the roller diameter (76 mm), while the contact width in the *x*-direction demonstrated a greater value due to the semi-circular profile of the raceway along that axis. The maximum contact pressure under the operation condition was about 3307 MPa, which occurred at the 14 mm position in the *x*-direction.

**Figure 6:**
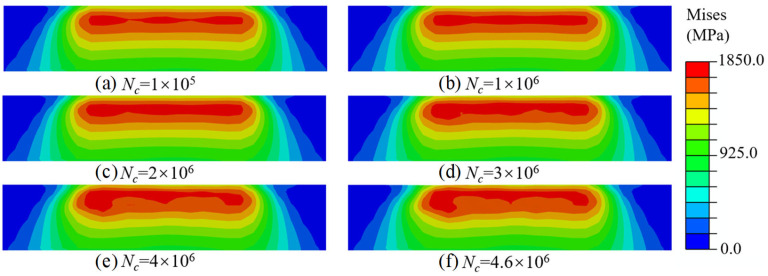
Evolution of maximum von Mises stress in the subsurface of the raceway.

**Figure 7:**
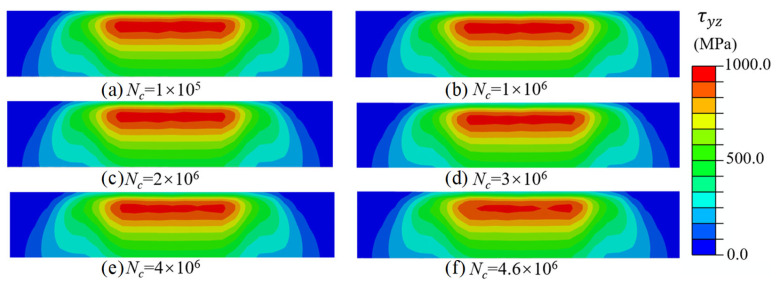
Evolution of shear stress range in the subsurface of the raceway.

Figure 6 and Figure 7 show the contours of maximum $\sigma_{eq}$ and Δτ throughout a complete loading cycle. It was found that the maximum values of $\sigma_{eq}$ and Δτ occurred at a subsurface depth beneath the raceway surface rather than at the contact surface. Theoretically, the value of maximum $\sigma_{eq}$ and Δτ should be identical at the same depth. However, the influence of friction force and simulation error leads to a slight variation in the stress response along the depth direction. Additionally, it was observed that the contours for both stresses did not change significantly during most of the fatigue lifetime. This is because the observed stress variation under a constant load amplitude is primarily attributable to the degradation of the raceway mechanical properties, whereas the degradation of material mechanical properties is very slow throughout the majority of the fatigue life cycle.

**Figure 8:**
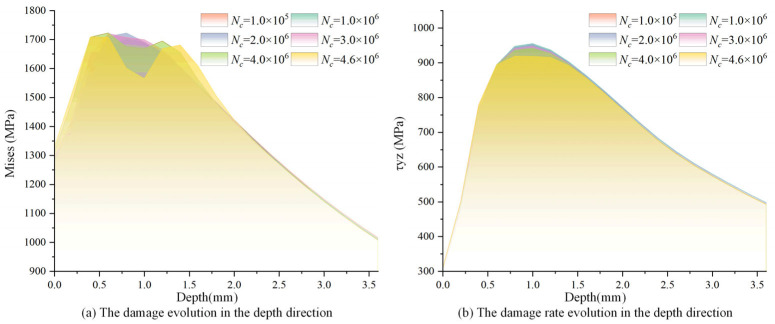
Evolutions of the maximum $\sigma_{eq}$ and Δτ in the depth direction for different loading cycles.

Figure 8 shows the evolution of the maximum $\sigma_{eq}$ and Δτ in the depth direction across varying loading cycles. Both maximum $\sigma_{eq}$ and Δτ gradually decreased as the loading cycle increased. Meanwhile, the reduction in stress was most significant near the critical element. For example, when the loading cycle increased from 1 to 4.6 million, the von Mises stress decreased from 1722 MPa in the initial state to 1601 MPa in the critical damage state for the critical element, with a 7% drop. Correspondingly, the shear stress range decreased from 947 MPa to 918 MPa, with a 3% drop. This is because the load-bearing capacity of material point is gradually weakened as the fatigue damage accumulates. Subsequently, the material stress in the critical point decreases.

### 3.2. Damage and Material Properties Evolution of Pitch Bearing

**Figure 9:**
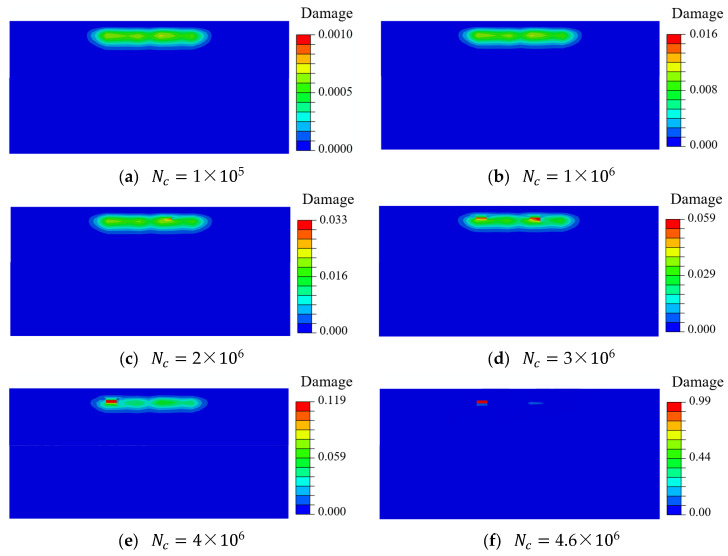
The evolution of subsurface damage.

**Figure 10:**
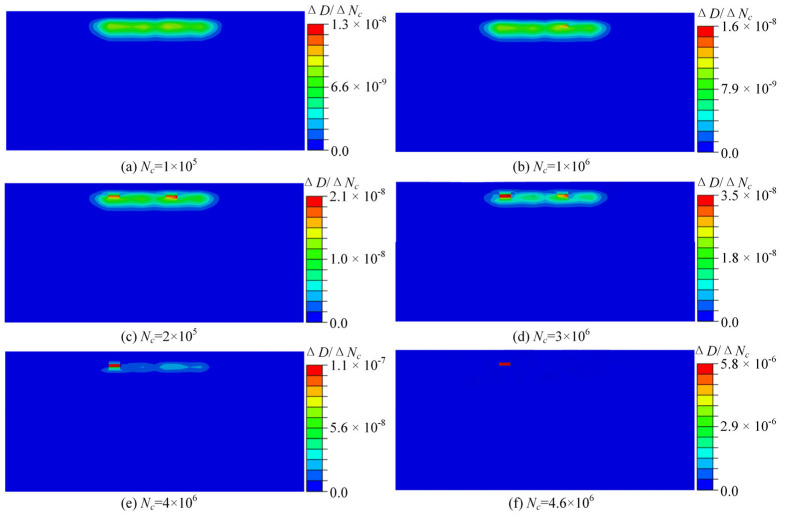
The evolution of damage rate.

Figure 9 shows the evolution of the fatigue damage contour under different numbers of loading cycles for the pitch bearing. The constant $\Delta N_{c}$ was set as 100,000. The accumulated damage under different loading cycles was concentrated near the region with the maximum von Mises stress and the shear stress range, and the damage of material points within this region increased as the loading cycle increased. The maximum subsurface damage was only 0.119 when the loading cycle reached four million. However, the maximum subsurface damage accumulated from 0.119 to the threshold value of unity after an additional 0.6 million loading cycles. This indicates that the accumulation of damage exhibits nonlinear characteristics, and the damage rate gradually accelerates. Figure 10 depicts the evolution of the damage rate as the loading cycle increased, the damage rate exhibited a high degree of similarity to the evolution of damage.

**Figure 11:**
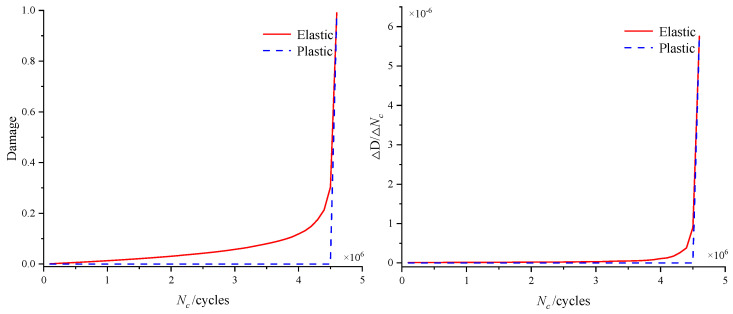
The evolution of elastic and plastic damage and damage rates.

The material element that first reaches the damage critical value is defined as the critical element. Figure 11 shows the evolution of elastic and plastic damage and damage rate of the critical element. It is revealed that both the elastic damage and damage rate grow exponentially. The elastic damage rate grew slowly in the early stage of rolling contact, which led to a slow accumulation of damage at the critical point. However, with the increase in the loading cycle, the material properties such as elastic modulus and damage parameters degraded continuously, resulting in a faster growth of the elastic damage rate. Hence, the damage rate increased sharply to the critical value in the last 10% of the remaining lifetime. In addition, it was found that the plastic damage was almost zero for most of the lifetime, indicating that no plastic deformation occurred under this load amplitude in the early stage; thus, no plastic damage accumulation was caused. It was revealed that the fatigue life of the pitch bearing was dominated by the elastic damage.

**Figure 12:**
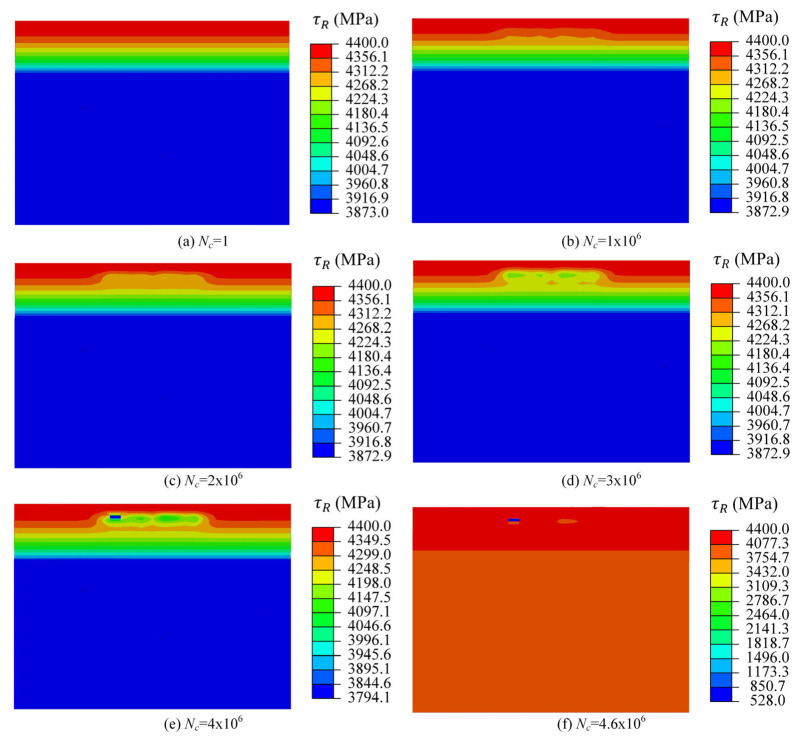
The evolution of elastic damage-related parameter $\tau_{R}$.

Figure 12 shows the evolution of elastic damage-related parameter $\tau_{R}$ as the loading cycle increased. $\tau_{R}$ is highly related to the material hardness; the hardness remains constant at the same depth, so $\tau_{R}$ is the same. $\tau_{R}$ gradually decreased from the maximum value of 4400 MPa on the surface to 3873 MPa in the core for the un-damaged raceway, as shown in Figure 10a. In addition, the evolution of $\tau_{R}$ represents the damage accumulation of material points. The damage of material point increased with the increase in the loading cycle, and further led to the deterioration of hardness, results in a decrease in $\tau_{R}$. For example, $\tau_{R}$ decreased from the initial maximum value 4400 MPa to the minimum value 528 MPa for the critical element when the loading cycle increased from 1 to 4.6 million.

**Figure 13:**
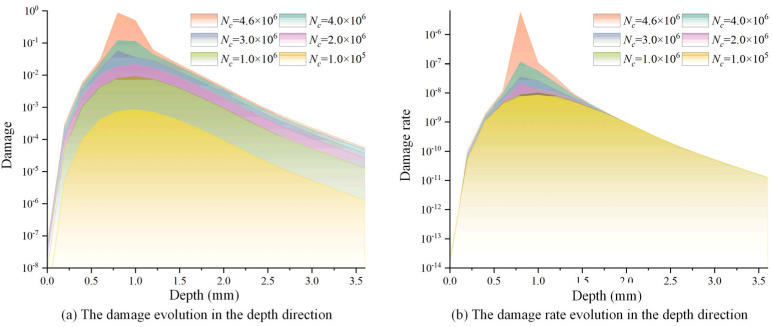
Evolutions of damage and damage rate in the depth direction for different number of cycles.

Figure 13 shows the evolutions of damage and damage rate in the depth direction for different loading cycles. Both the damage and damage rate first increased to the maximum at 0.80 mm and then decreased as the depth increased. When the loading cycle reached 4.6 million times, the damage rate depicted a sudden growth in the depth of 0.8 mm, and the cumulative damage reached the critical value of unity meanwhile, leading to the fatigue failure of the bearing raceway.

Table 3 lists the evolution of the critical element damage-related parameters. The accumulated damage was less than 0.12 in the first 90% fatigue lifetime. The von Mises stress was 1682.8 MPa in the initial loading cycle, which was lesser than the initial yield stress. Hence, the total damage was also the elastic damage because the PEEQ was zero. The yield stress decreased due to the accumulation of damage after 2 million loading cycles, which resulted in the occurrence of plastic strain and plastic damage. In addition, the maximum von Mises for the raceway was 1775 MPa, which is not in the position at which the fatigue failure first occurs. It is concluded that the fatigue failure of raceway first occurs at the position with the maximum shear stress range rather than the one with the maximum von Mises stress.

## 4. Conclusions

The rolling contact fatigue (RCF) performance of pitch bearing raceways plays a critical role in ensuring the structural integrity, operational reliability, and extended service life of offshore wind turbine systems. To address this challenge, this study developed a continuum damage mechanics (CDM)-based computational framework for simulating RCF behavior in pitch bearings under operational conditions. The proposed model integrated damage-coupled constitutive equations with a progressive damage evolution law, rigorously formulated to quantify the gradual deterioration of the material mechanical properties under cyclic loading. The fatigue process was simulated through using both a user subroutine UMAT and a Python script. The principal conclusions derived from this investigation can be summarized as follows:

1. Under the rated operational condition, the pitch bearing raceway exhibited a peak contact pressure of 3307 MPa; the corresponding maximum von Mises stress was 1775 MPa within the critical subsurface layer at 0.8 mm depth.
2. The fatigue damage evolution demonstrated nonlinear accumulation patterns, in which the cumulative damage remained below 0.12 throughout the initial 90% of the fatigue life cycle.
3. The fatigue failure of the raceway initially occurred at the position with the maximum shear stress range rather than at the position with the maximum von Mises stress.
4. Elastic damage accumulation served as the predominant governing factor in determining the operational fatigue lifespan under operational cyclic loading conditions.

Additionally, there are some limitations in this study. First, the influence of friction coefficient variations and service temperature fluctuations was not addressed. Second, the finite element simulation neglected the dynamic interactions between adjacent, mobile bearing components and their collective impact on contact mechanics and load distribution patterns. These simplifications may introduce deviations from real-world operational conditions. Future work should incorporate parametrization studies to quantify these effects.

## Figures and Tables

**Table 1:** Chemical composition of the pitch bearing raceway 42CrMo4.

C	Si	Mn	Cr	P	S	Mo
0.40	0.30	0.75	1.05	0.01	0.004	0.25

**Table 2:** Material damage parameters for pitch bearing.

m	12.09	q	6.05
$\tau_{R(Sur)}$	4806.45 MPa	$s_{\left(Core\right)}$	28.60 MPa
$\tau_{R(Sur)}$	3018.25 MPa	$s_{\left(Core\right)}$	11.28 MPa

**Table 3:** Evolution of the critical element damage-related parameters.

*N* (×10^5^ Cycles)	Damage	Damage Rate	von Mises (MPa)	Shear Stress Range (MPa)	PEEQ
1	0.0008	7.51 × 10^−9^	1682.8	918.5	0
10	0.0081	8.84 × 10^−9^	1702.9	947.4	0
20	0.0190	2.06 × 10^−8^	1722.3	944.1	1.07 × 10^−5^
30	0.0579	3.53 × 10^−8^	1708.0	940.7	2.30 × 10^−4^
40	0.1187	1.13 × 10^−7^	1675.0	936.3	7.28 × 10^−4^
46	0.9913	5.76 × 10^−6^	1601.1	918.5	1.43 × 10^−2^

## Data Availability

The raw data supporting the conclusions of this article will be made available by the authors on request.
